# Better Lives for Dogs: Incorporating Human Behaviour Change Into a Theory of Change to Improve Canine Welfare Worldwide

**DOI:** 10.3389/fvets.2018.00093

**Published:** 2018-05-28

**Authors:** Karen Reed, Melissa M. Upjohn

**Affiliations:** ^1^Executive Director, Dogs Trust Worldwide, London, United Kingdom; ^2^Canine Behaviour and Research, Dogs Trust, London, United Kingdom

**Keywords:** canine welfare, theory of change, human behavior change, non governmental organisation, international

## Abstract

The world’s estimated 600 million dogs face a range of welfare issues which vary according to local context and locally accepted norms regarding attitudes towards dogs. Dogs Trust Worldwide, an international Non-Governmental Organisation which works to improve canine welfare, is applying a Theory of Change framework to define and unpick key challenges faced when collaborating with local partners to achieve its mission. We describe the Theory of Change approach and the importance of Human Behaviour Change within this. We identify questions which need to be addressed as part of articulating our ways of working with partner organisations and acknowledge issues around generating evidence to measure the impact our work has on the ultimate beneficiaries.

## Introduction

Reliable figures for the worldwide dog population are difficult to source. Available estimates include the World Society for Protection of Animals’ figure of combined stray and owned animals of 600 million (1). Based on studies published by Non-Governmental Organisations (NGOs) working worldwide with other species (2), it may be assumed that welfare issues affecting dogs vary around the world; it could be proposed that they are influenced by ownership patterns, availability and uptake of canine healthcare and welfare facilities and locally accepted norms regarding attitudes towards dogs. However, these theories need to be tested empirically.

Dogs Trust is a canine welfare NGO whose mission is to bring about the day when all dogs can enjoy a happy life, free from the threat of unnecessary destruction. While its original remit focused on improving United Kingdom-based canine welfare, as wider challenges affecting dog welfare are recognised the organisation increasingly works with partner organisations around the world to address these. Dogs Trust Worldwide (DTW) works directly through DTW staff in Bosnia and Herzegovina but also supports many partners through sharing expertise and experience and providing grant funding. The focus is on 5 priority areas : humane dog population management; rabies prevention; supporting veterinary care; rehoming and stopping exploitation, all underpinned with education and community engagement interventions. The complexity and interconnectedness of these areas, plus working at a distance through multiple, diverse partners (ranging from small, largely volunteer run organisations to large non-profits working in more than one country) represent some of the challenges faced.

As part of reviewing its strategic approach to international work DTW is applying a Theory of Change (ToC) framework to define and unpick some of the key challenges by spelling out the processes and assumptions embedded within them. Learning from this review will inform design of its ways of working with partners in particular, and improved ways of monitoring achievements. This paper summarises how a ToC framework informs the process which DTW is following and highlights some of the key issues which are being considered in working towards achieving effective resource allocation and measuring progress towards improved canine welfare.

## What Is a Theory of Change?

NGOs are typically established to address a social issue and/or to deliver resources at a local, national or international level. Regardless of scale, every NGO needs a clear understanding of the social and practical context within which its activities are set, how its activities contribute towards achieving the desired situational change, how they inter-relate with activities of other stakeholders and what assumptions underlie its ways of working towards achieving that change.

The NGO sector adopts ToC terminology in multiple contexts to articulate this understanding. As noted by Vogel and Stephenson (3) “Some people view [ToC] as a tool and methodology to map out the logical sequence of an initiative from inputs to outcomes. Other people see it as a deeper reflective process and dialogue amongst colleagues and stakeholders, reflecting on the values, worldviews and philosophies of change that make more explicit people’s underlying assumptions of how and why change might happen as an outcome of the initiative”. ToC is best viewed as a flexible approach, comprising “Theory of Change thinking” rather than as a rigid tool or methodology.

A ToC should be as comprehensive a representation as possible of all the pathways that may lead to change, even those in which the NGO is not involved. The ToC must clarify components over which the NGO has control, those for which it relies on behaviour of and contributions from third parties and those which are driven by external factors such as social, cultural and environmental constraints. It must describe how and why each change happens. Components involving third parties require making assumptions about the causal pathway that underlies others’ actions and the motivations and wider societal factors that drive or influence behaviour change and/or societal norms (4). According to Vogel (5) “…assumptions act as “rules of thumb” that influence our choices, as individuals and organisations. Assumptions reflect deeply held values, norms and ideological perspectives. These inform the design and implementation of programmes. Making assumptions explicit, especially seemingly obvious ones, allows them to be checked, debated and enriched to strengthen programmes.”

ToC focuses on wider societal changes that are being targeted (results) and how they come about, rather than solely on the programmes and services that the organisation delivers. However, an organisation may use a ToC at project level to identify where their accountability ends or what the accountability ceiling is and it can help to identify if assumptions above that level justify the project.

If well used, the ToC can enable the organisation and its partners to critique and challenge structural approaches with a view to understanding whether they deliver the results that are being targeted. If expected results are not being delivered, the organisation can consider how to improve effectiveness by modifying its own activities, reconsidering the inherent assumptions and/or adapting its interactions with other stakeholders. An example of points that can be reviewed in this way is described by Liszewski (6), where the preconditions that are assumed for changing human behaviour in relation to the welfare of working equids are Capacity, Motivation and Opportunity.

## Challenges Encountered in Applying Theory of Change

According to Brown (7) the steps required to create a ToC are as follows:

- Identify a long-term goal
- Conduct “backward mapping” to identify the preconditions necessary to achieve that goal
- Identify the interventions that will be performed to create these preconditions (outcomes)
- Develop indicators for each outcome that will be used to assess the performance of the interventions
- Draft a narrative that can be used to summarise the various components of the ToC

The author described ToC as having the following components: Outcomes, modelled in causal pathways; Interventions (activities) leading to the relevant outcome(s); Assumptions; Rationales; Indicators and Narrative.

Conducting the “backward mapping” may reveal that the preconditions for success are not a clearly defined, linear process. Interactions of multiple stakeholders combined with environmental challenges led Ellerman (8) to describe “wicked” problems in social, economic and political contexts as being characterised by “novel complexity, genuine uncertainty, conflict of values unique circumstances and structural instabilities”. In some situations, improving canine welfare may be found to be an example of this kind of problem.

(9, 10) noted that a ToC is often complex and involves mutual influence, parallel processes and feedback loops. He proposed that a network approach to understanding interactions between stakeholders may be better than the traditional linear Logical Framework (logframe) representation as there may be situations where the ToC is more complex.

The format in which a ToC is presented should take account of its target audience or audiences. There may be multiple audiences; lay supporters or others with limited time to assimilate technical details may prefer less complexity whereas specialist staff or institutional donors may require a more complex version, so multiple, factually consistent versions with varying levels of detail may be required. A diagram with a narrative text is usually provided but there is no standard for the format for the diagram or its components. The narrative should include descriptions of why one box will lead to another box. If assumptions have been made about how certain stakeholders will act within a chain of events, evidence to support that needs to be described e.g., if you think increased knowledge will lead to behaviour change, is that an assumption or do you have evidence to show it is the case?

Organisations such as the United Nations advocate the use of Results Based Management (RBM) as a strategic management approach which operationalises ToC within planning and feedback-based monitoring (11).

**Figure 1:**
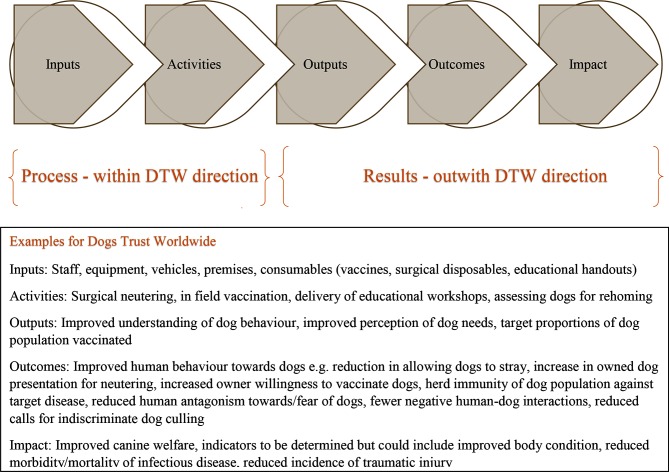
Results Based Management Framework as it relates to Dogs Trust Worldwide.

Figure 1 illustrates a simplified model of the phases of a causal pathway that is typically used as part of an RBM framework. It also summarises key example components for DTW across several work themes.

Stern et al. (12) noted in their report about monitoring NGO interventions that “most interventions are “contributory causes”. They “work” as part of a causal package in combination with other “helping factors” such as stakeholder behaviour, related programmes and policies, institutional capacities, cultural factors or socio-economic trends.” They also noted that “…when assessing impact of an intervention it’s important to ask “Did the intervention make a difference?” which allows space for combinations of causes rather than “Did the intervention work?” which implies an intervention is a cause acting on its own.” This distinction between recognising contribution rather than assuming attribution means that caution may be advised in the use of RBM-style frameworks for planning and monitoring. The effect of external influences on results needs to be explicitly integrated within the reporting format, rather than presuming a direct link between activities, outputs, outcome and impact and simply listing assumptions, as in RBM.

## Issues Affecting Application of Theory of Change to Animal Welfare Interventions

As noted by Ellerman (13) “most real assistance work is concerned with deeper changes of (human) culture change, capacity building and sustainability...”. This involves respecting the autonomy of the doer (13) and “ensuring that programme content... is constituted and organised by the students” view of the world’ (14).

Many animals’ welfare status is largely determined by human-managed activities and attitudes; populations of domesticated species are generally unable to facilitate welfare improvement for themselves (15). Interventions targeting sustainably improved animal welfare therefore almost invariably rely on achieving human behaviour change (HBC). The challenge is to understand the root cause of those situations and articulate a pathway to facilitate HBC for the benefit of animals, rather than to benefit humans.

For example, is a human welfare benefit essential in order to incentivise HBC for animal welfare benefit? What if there is a human welfare cost (real or opportunity) associated with improving animal welfare? What if there is a short-term welfare cost for certain individual animals to trade off against a long-term welfare benefit for a population of dogs? Utilitarianism, articulated by Bentham in the late 18th century, proposes that the best action is the one that maximizes “utility” defined in terms of the well-being of sentient beings. How is the “value” of animal welfare quantified for comparison with human welfare to calculate this? Deontology involves judging the morality of an action based on rules, which themselves may range from ethical naturalism, religious law or personal values (16). (How) does this theory explain people’s opinions and decision making relating to animals?

Different cultures have widely varying views of animal welfare and the recognition of sentience in different species. In depth consultation with local stakeholders about the basis of these views and potential routes to understanding these perspectives is essential before any attempt to address their impact on animal welfare can be considered. Situational analysis, intervention design and implementation should be undertaken by animal owning communities and policy makers themselves to maximise the chances of engagement in achieving desired behaviour change (17). These processes will need to take account of any heterogeneity of ownership characteristics (owned as pets, working animals, farmed animals, strays/feral) within the same species.

Depending on the context and local norms, it may be useful to encourage people to put themselves in the animal’s situation to appreciate the welfare need and how that is linked with HBC (18). “Speciesism” may result in variable recognition of sentience or welfare needs affecting attitudes and motivations to address poor welfare in different animals. This may even apply within species; in South Korea there is a common belief that there are two types of dogs: pet dogs for companionship and meat dogs for human consumption who are considered “soulless” (CFAF, undated). In some developing countries community discussions have noted that “donkeys don’t get sick or if they do, they always die so there’s no need to invest in their health needs” (personal communication, Kimberly Wells). Any intervention needs to be designed to address these differing perspectives, social values and potential motivations and taking into account assumptions around the reasons and/or drivers for the desired change to happen.

Gathering data to verify the ToC in relation to animal welfare interventions can be challenging, and there are few peer reviewed published impact assessment reports (19). Much of the process involves measuring changes in peoples’ attitudes or behaviours toward animals; generating objective measures of these indicators is challenging since a subject’s behaviour may change if they are aware of being observed; similarly, their response to a questionnaire may be influenced by what they think the interviewer wishes to hear. Sourcing objective data from the animal to evidence human behaviour may also be difficult. Informed consent from third parties may also be required to gather data and robust data storage protocols need to be established to ensure data security compliance.

## Developing a Theory of Change for Canine Welfare

**Figure 2:**
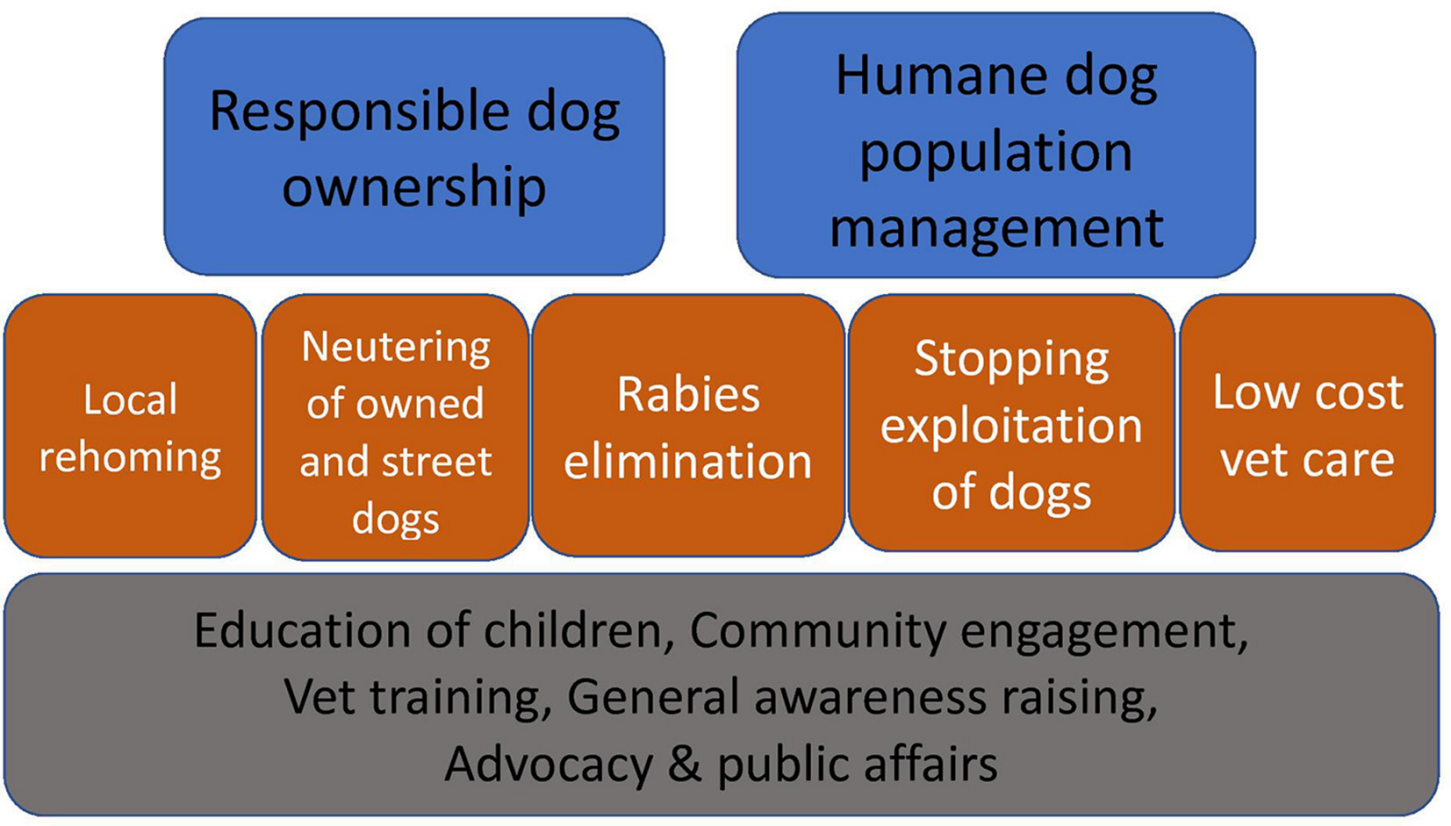
Dogs Trust Worldwide Theory of Change Framework.

Figure 2 illustrates the overall ToC framework that DTW is in the process of developing, through in-house workshops with programmatic and technical teams, both in the UK and in Bosnia. It illustrates how DTW’s target goals of responsible dog ownership and humane dog population management are based around supporting five work themes, described earlier. Each of these themes may be addressed in many countries. Narrative to support this diagram by explaining the assumptions underlying it and documenting the evidence to confirm these or gaps therein is currently being developed by DTW staff in discussion with programmatic partners and wider Dogs Trust teams. The nature of the multiple and diverse partnerships existing with DTW has precluded the involvement of some key stakeholders in this process, which is a challenge that needs to be addressed. Having this representation needs to be factored in to the ongoing development of the ToC to add to and help test some of the key assumptions being made.

**Figure 3:**
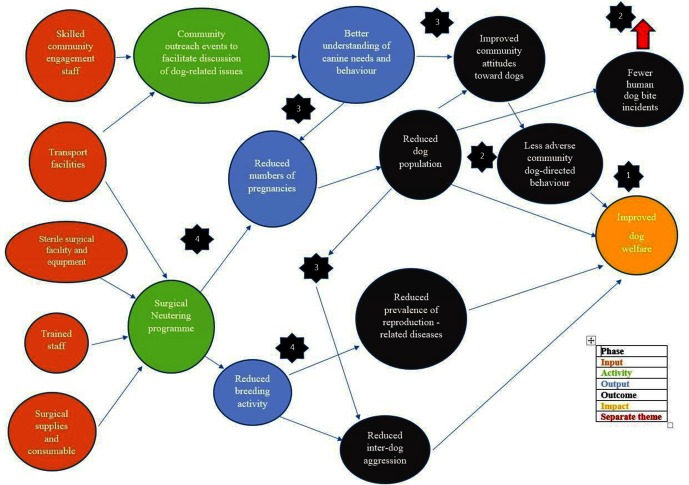
Process for Dogs Trust Worldwide Dog Population Management theme.

DTW is developing a definition of process for each of the five themes described earlier as a prelude to identifying the inter-relationships between the themes as part of articulating these assumptions. A separate process map for each of the five themes is in preparation by DTW staff. Figure 3 shows an illustrative example of the process map for the Humane Dog Population Management (DPM) theme including associated community outreach activities. Each phase of the process is colour coded according to the key shown. DTW recognises that rabies links human health management with human attitudes and interactions with dogs in countries where the disease is endemic. This means that the DPM theme is inextricably linked with human health management processes, even though those processes are outwith DTW’s remit. This link is indicated within the process map by the red arrow.

In developing the DTW ToC a series of questions arise. Those which need to be addressed in the worked process example in Figure 3 are indicated with the number corresponding to the item shown in the figure. The questions are listed in Table 1, along with brief details of whether they have been resolved and if not, next steps to achieve this. They are divided into programmatic, logistical and performance management and reporting topics.

**Table 1:** Questions arising during the development of Dogs Trust Worldwide’s Theory of Change Items that are illustrated for Dog Population Management theme in Figure 3 are annotated by a star and a number to indicate the relevant step.

**Question**	**Progress**
**Programmatic topics**	
What is DTW’s desired impact? What indicators are most suitable to measure impact?	The DTW team has defined impact as improved canine welfare. Potential indicators of canine welfare for each theme are under development by DTW team in conjunction with partners and wider Dogs Trust departments including Education and Research teams.
Is it necessary to distinguish owned, non-owned and shelter dogs as separate target groups within each theme and overall ToC?	There is an assumption that these groups do need to be considered separately within each theme.As outcome indicators under each theme are addressed, the need to retain these groups as discrete populations or not will become clearer.
What outcomes immediately precede impact and how do these outcomes inter-relate? Which involve DTW and which involve other agencies? What assumptions underlie the link between each outcome and impact? *1	Definition of outcomes and the parties involved in their achievement are under development by the DTW team as part of drafting a process map for each theme. The parties involved will be consulted to confirm availability of evidence to inform assumptions and to identify evidence gaps that need to be investigated.
(How) does the ToC recognise linkages between improving canine welfare and changes in human welfare? How can/are these be monitored/measured? For the international context, examples of linkages could include: canine rabies vaccination programmes to reduce incidence of canine rabies, reduction in human rabies incidence, changes in human perceptions of threat posed by/attitudes towards free roaming dogs *2 education programmes to improve people’s understanding of how to be safe around dogs e.g., appreciating dog behaviour and how managing your own behaviour, reduced adverse human-dog interaction/dog bite incidence, reduction in incidences of dog aggression and potential changes in fear of dogs *3 DPM (neutering) programmes to reduce number of puppies being born and thereby reduce competition for food, reduce risk of mating related inter-dog aggression for males, reduce pregnancy/lactation associated welfare issues for females, changes in human perceptions of the nuisance/threat posed by/attitudes towards free roaming dogs *4	Linkages for the DPM theme have been identified as part of drafting Figure 3, see red arrow.Indicators to measure and monitor these are under consideration, taking into account availability and reliability of existing data from other sources.
How are “responsible” and “ownership” defined within Responsible Dog Ownership (RDO)? (How) do these definitions vary in different countries or different regions?	Indicators of responsible dog ownership in the UK will be used initially to test out the assumptions that some of these can be generalised across different contexts.
How comparable is the HBC that we’re seeking in different contexts?	There is an assumption that the long term HBC may be similar but that milestones will differ.
How do outcomes and impact vary across different countries with substantially varying canine welfare contexts and prevalent issues?	There is an assumption that the desired impact remains the same across all projects but that outcomes may vary in relative importance.
**Logistical topics**	
How does DTW select partners? Is DTW willing to/actively seeking to work with organisations who primarily target human welfare issues rather than canine welfare issues?	Further internal discussions will be needed to develop consensus.
Depending on the elements of the ToC we focus on, how does the interaction of the various elements of the ToC impact selection of partners, programme design and implementation of monitoring?	Strategically, having a wide selection of large and small partners across all themes will be required.
How do partners’ different objectives affect feasibility of collaborative working opportunities, prioritisation of resource allocation? Per Gasper (20) an assumption of consensual project objectives may become problematic in inter-organizational projects. Similarly, Jones (21) described “Implementation cannot be technocratic but requires a negotiated understanding and synthesis through communicative processes”.	It is likely that longer term partners who have been involved with DTW for some time and who can understand ToC thinking will be more able to work collaboratively on this.
How does DTW balance addressing (potentially emotive) short term welfare problems that our supporters may expect us to fix via, for example, provision of emergency veterinary services, whilst risking potentially creating “dependency” or crowding out local solutions, with targeting long term sustainable welfare improvements that require more complex approaches? How are these different approaches incorporated within the ToC?	There is an assumption that the balance of small and large partners, some of whom do have a similar understanding as DTW of targeting sustainable welfare improvements will be important to support those differing expectations.
How does provision of “free” and subsidised services such as neutering programmes/vaccination programmes interact with owners’ motivation to engage with RDO practices and with governments’ recognition of their need to deliver “public health” services?How does this affect long term sustainability of programmes to improve dog welfare?	DTW is targeting key partners who can work with DTW on this, whilst allowing organisation with currently different thinking but the ability to change to also be supported.
**Performance management and reporting**	
How can data be collected to measure the various elements of the ToC, whether those in which DTW is directly involved or others?	Some larger partners already do collect good data and have the ability to collect wider data going forwards.
How are target levels for outputs/outcomes/impact set across different countries?	This is currently done in negotiation with individual grant holders. Thus there is no consistency/standardisation but frameworks will be worked on during 2018.
How good is “good enough” in terms of our target welfare standards in different contexts?	Discussions are required to achieve internal consensus on this.
Is “good enough” different in different places, recognising that there may be external factors that we can never realistically hope to influence?	Milestone setting with partners will aim to address this.
Can measures for outputs/outcomes/impact be aggregated across different contexts?	DTW will aim to try to identify some key indicators that can be aggregated.

The multiple, as yet, unanswered questions in the table will direct the focus of the work to continue development of this ToC including: developing an internal consensus on desired outcomes; informing the stakeholder involvement required; addressing the need for clarity on definitions; the careful consideration of variable contexts and the differing expectations of stakeholders.

## Conclusion

The theory of change for improving canine welfare internationally is a “complex” or “wicked” problem. Whilst the outline high level framework for DTW’s ToC has been drafted, process maps for each theme of DTW’s work now need to be completed. The narrative to underpin the framework and to explain the assumptions embedded within each theme can then be developed. This will focus particularly on understanding the importance of HBC to the process and articulating the assumptions that have been made around what influences this change and how best change can be facilitated. Participatory approaches need to be employed to test and gain insight into underlying assumptions. This will enable us to ensure that, as much as possible, donor aims and human recipient desires align for the maximum benefit of canine welfare.

## Author Contributions

Both authors co-conceived the topic for the paper, MMU drafted the original text and incorporated changes proposed by KR to produce final version of the paper for submission.

## Conflict of Interest Statement

KR is employed by Dogs Trust Worldwide. MMU is employed by Dogs Trust.
